# Recurrent spontaneous pneumothorax in young woman: catamenial pneumothorax

**DOI:** 10.11604/pamj.2016.23.44.8877

**Published:** 2016-02-18

**Authors:** Adriá Rosat, Jorge Herrero

**Affiliations:** 1 Department of General Surgery, Hospital Universitario Nuestra Señora de Candelaria, Ctra, Del Rosario 145, 38010 Sta, Cruz de Tenerife, Spain; 2 Department of Thoracic Surgery, Hospital Universitario Nuestra Señora de Candelaria, Ctra, Del Rosario 145, 38010 Sta, Cruz de Tenerife, Spain

**Keywords:** Recurrent, spontaneoous, pneumothorax, catamenial

## Image in medicine

A 31-old-year woman came to hospital for dyspnea. She was a non smoker patient and had no history of previous pneumothorax. She was under gynecologic study for having troubles to get pregnant. On physical examination she had tachypnea and right chest hypoventilation. Blood test showed slight leukocytosis and chest x-ray revealed a right hemopneumothorax. A chest tube was placed and few days later she required surgery for persistent air leak. Apical blebs were found and resected by video thoracoscopy. Gynecologic studies were completed and she was diagnosed of pelvic endometriosis and treated by bilateral salpingectomy and left partial oophorectomy with confirmed endometriosis on pathology. She had three more episodes of smaller right pneumothorax in relation with menses. On the last episode she underwent surgery to perform a pleurodesis. Small tissue implants were resected, which were negative for endometriosis. One year later she has had no more episodes and she is actually under in vitro fertilization procedures to get pregnant. Catamenial pneumothorax is related with up to 3% of spontaneous pneumothorax in women, and should be considered as differential diagnosis, especially in those with troubles to get pregnant.

**Figure 1:**
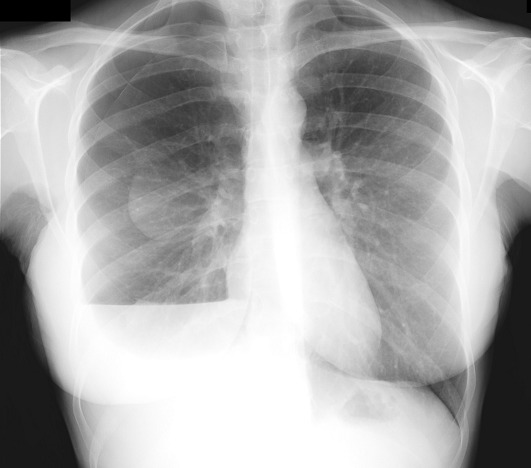
Right chest hemopneumothorax

